# Use of the Endoscopic Healing Index for Monitoring of Disease Activity in Patients With Crohn’s Disease in the COVID Era

**DOI:** 10.1093/crocol/otaa035

**Published:** 2020-05-02

**Authors:** Maria T Abreu, Lauren Okada, Thierry Dervieux, Allison Luo, Anjali Jain, Timothy Ritter, Stephen B Hanauer

**Affiliations:** 1 Division of Digestive Health & Liver Diseases, University of Miami Miller School of Medicine, Miami, Florida, USA; 2 Prometheus Biosciences, San Diego, California, USA; 3 GI Alliance Research, Southlake, Texas, USA; 4 Northwestern University Feinberg School of Medicine, Chicago, Illinois, USA

***To the Editors***,

Coronavirus disease 2019 (COVID-19), caused by the SARS-CoV-2, is projected to infect 40%–70% of the population.^1^ C-reactive protein (CRP) both correlates with and predicts COVID severity.^2, 3^ Clinical experience from China and Singapore has reported elevated CRP concentrations in COVID-19-infected patients (China: ~60% patients with CRP ≥10 mg/L, Singapore: median [range] = 16.3 mg/L [0–97.5]).^4, 5^ In the absence of universal COVID testing, use of CRP as a biomarker for the assessment of disease activity in patients with Crohn’s disease (CD) who may also be infected with COVID could be inaccurate. Measuring fecal calprotectin may also pose safety concerns associated with collection and handling of fecal material, which has a high SARS-CoV-2 viral RNA concentration.^6^

The Endoscopic Healing Index (EHI, Monitr), a serum test that combines 13 protein biomarkers to produce a quantitative EHI score (range 0–100), was developed and validated against colonoscopy in patients with CD.^7^ At a cutoff <20, EHI yields an 83.2% sensitivity to rule out endoscopically active disease; while a cutoff >50 yields an 87.8% specificity to rule in endoscopically active disease. We tested the hypothesis that varying the levels of CRP, one of the proteins measured in the EHI assay, would not significantly change the proportion of patients with CD identified as having active disease.

To test this hypothesis, we performed an in silico experiment to assess the impact of isolated CRP elevation on EHI score and its categorical assignment. De-identified results from 14,284 EHI tests (from 11,760 patients, median age [IQR] = 44 years [29.5–60], 57.8% females) were selected from a CAP-accredited clinical laboratory database (Prometheus Biosciences, San Diego, CA). Median [IQR] of EHI was 32 [20–46]. CRP concentrations in specimens with an original CRP ≤5 mg/L (8238 specimens, median [IQR] = 1.4 mg/L [0.5–2.8]) were systematically replaced with concentrations ranging from 10 mg/L up to the upper limit of quantitation of the assay [60 mg/L]. Simulated EHI scores for each specimen were then recalculated by substituting the original CRP values with the hypothetical CRP concentrations while keeping the concentrations of the other 12 markers constant.

The percentage of specimens in the clinically relevant groups of EHI <20, EHI 20–50, and EHI >50 was 33.2% (N = 2732), 59.2% (N = 4876), and 7.6% (N = 630), respectively. Median change in EHI score in specimens within the original EHI <20 group varied from 1 to 3 EHI units when CRP concentrations were increased from 10 to 60 mg/L (Fig. 1). The impact of increasing CRP levels on EHI categorical assignment (Table 1) revealed that 84.6% specimens presenting with EHI <20 remained within that group in the presence of several-fold increased CRP to 10 mg/L (median increase: 2 EHI units, IQR: [1–3]). A very large increase in CRP (to 60 mg/L) resulted in 31.8% specimens changing the EHI assignment from <20 to ≥20 but even this large increase in CRP concentration did not result in EHI >50.

**TABLE 1.** (A–D) Contingency Table of Numbers and Percentage of Specimens in Each EHI Group Using Simulated CRP Concentrations of 10 (A), 20 (B), 40 (C), and 60 (D) mg/L

	Original EHI: <20	Original EHI: 20–50	Original EHI: >50
**A Simulation: CRP = 10 mg/L**			
Simulated EHI: <20	2311 (84.6%)	0 (0%)	0 (0%)
Simulated EHI: 20–50	421 (15.4%)	4425 (90.8%)	0 (0%)
Simulated EHI: >50	0 (0%)	451 (9.2%)	630 (100%)
Total	2732	4876	630
**B Simulation: CRP = 20 mg/L**			
Simulated EHI: <20	2128 (77.9%)	0 (0%)	0 (0%)
Simulated EHI: 20–50	604 (22.1%)	4178 (85.7%)	0 (0%)
Simulated EHI: >50	0 (0%)	698 (14.3%)	630 (100%)
Total	2732	4876	630
**C Simulation: CRP = 40 mg/L**			
Simulated EHI: <20	1972 (72.2%)	0 (0%)	0 (0%)
Simulated EHI: 20–50	760 (27.8%)	3912 (80.2%)	0 (0%)
Simulated EHI: >50	0 (0%)	964 (19.8%)	630 (100%)
Total	2732	4876	630
**D Simulation: CRP ≥ 60 mg/L**			
Simulated EHI: <20	1862 (68.2%)	0 (0%)	0 (0%)
Simulated EHI: 20–50	870 (31.8%)	3738 (76.7%)	0 (0%)
Simulated EHI: >50	0 (0%)	1138 (23.3%)	630 (100%)
Total	2732	4876	630

Simulated EHI compared to EHI groups with original CRP concentrations (Original EHI). Percentages of simulated EHI groups are presented as column percentages calculated as a percentage of the original EHI group.

**FIGURE 1.**
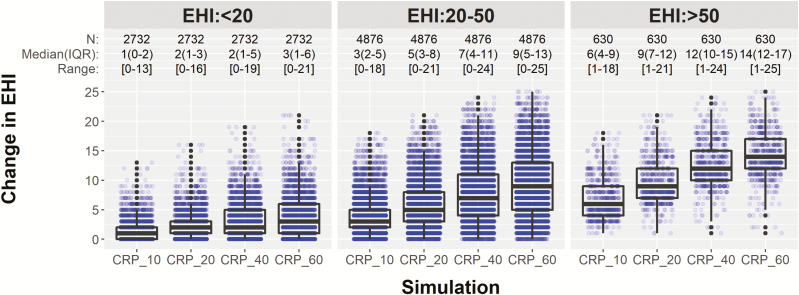
Change in EHI for each simulation grouped by original EHI scores. Within each EHI group, change in EHI at CRP concentrations of 10, 20, 40, and 60 mg/L is shown. Labels in each EHI group are listed in the following order as N (samples), median (IQR) of change in EHI, and [Range] of change in EHI.

Shelter at home and social distancing have been the primary measures used to control the spread of COVID-19. Most elective endoscopic procedures have been stopped as part of this effort. There is an urgent unmet need for safe and accurate noninvasive alternatives to endoscopy for assessing objective disease measure in patients with CD. Because CRP is a systemic inflammatory biomarker, it may not be reliable for measuring disease activity of CD in patients who may also have COVID-19 infection. In particular, gastrointestinal symptoms frequently occur with COVID-19 and may lead to confusion about IBD flare versus COVID-19.^8–10^

Our simulation data from 8238 patient specimens demonstrate that EHI scores remain relatively stable despite increased CRP concentrations, thus suggesting that even though CRP is one of the 13 markers within EHI, an isolated increase in CRP does not significantly impact the score or its clinical interpretation. Results from this simulation exercise are not unexpected as the biomarkers within the EHI algorithm are weighted, that is, the contribution of each biomarker to the algorithm is different. Therefore, the algorithm is not dependent on any one biomarker such as CRP but the EHI score is the result of all 13 biomarkers. In summary, EHI is potentially a reliable and robust alternative for the noninvasive assessment of objective disease activity in patients with CD.

## Notes

Conflicts of Interest and Financial Disclosures: MTA: Consultant for Janssen, Prometheus Biosciences, Takeda, Focus Medical Communications, Boehringer Ingelheim Pharmaceuticals, Gilead, Imedex, Cornerstone Health, Inc., Landos Biopharma, UCB Biopharma SRL, Eli Lilly, and Cosmo Biopharma; has received grant or research support from Prometheus Biosciences, Takeda, and Pfizer. TR: Served as a speaker, consultant, or advisory board member for Abbvie, Arena Pharmaceuticals, Boehringer Ingelheim, Celgene, Eli Lilly, Ferring, Genentech, Gilead, Ironwood, Janssen, Pfizer, Prometheus Biosciences, and Takeda. SBH: Consultant for Prometheus Biosciences. LO, TD, AL, and AJ are employees of and own stocks and/or stock options for Prometheus Biosciences.
